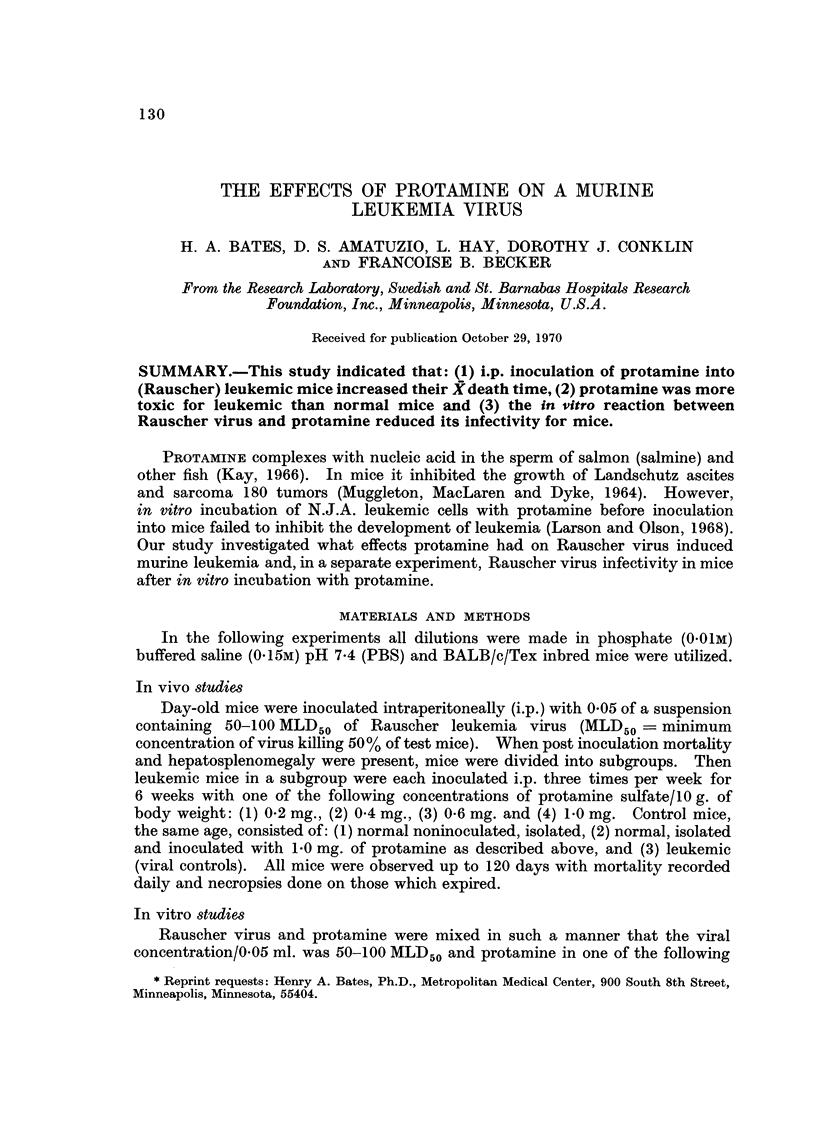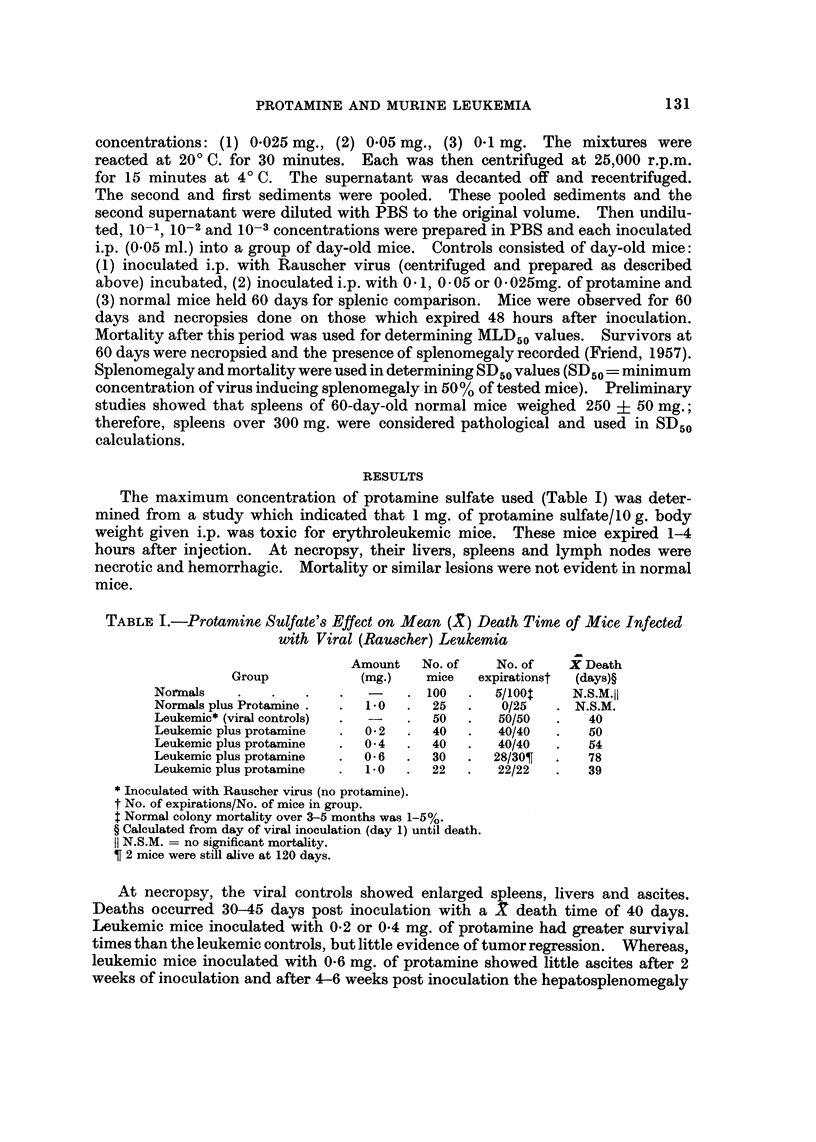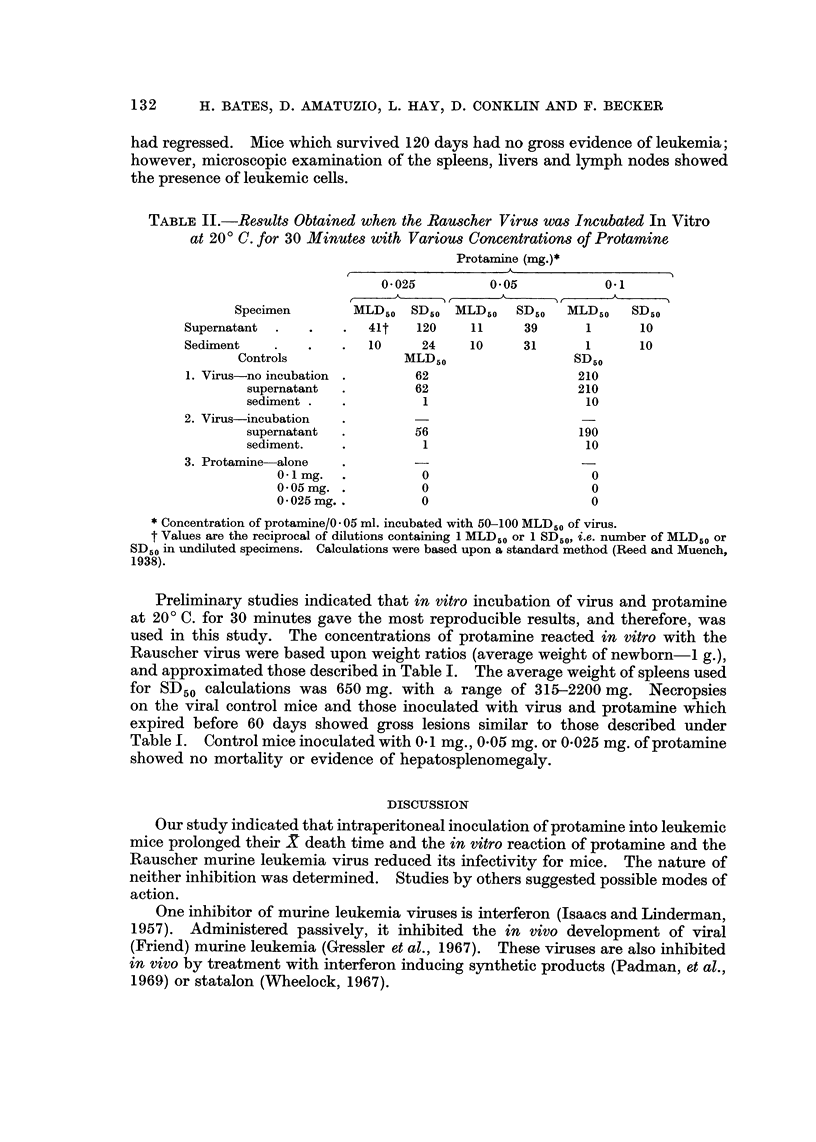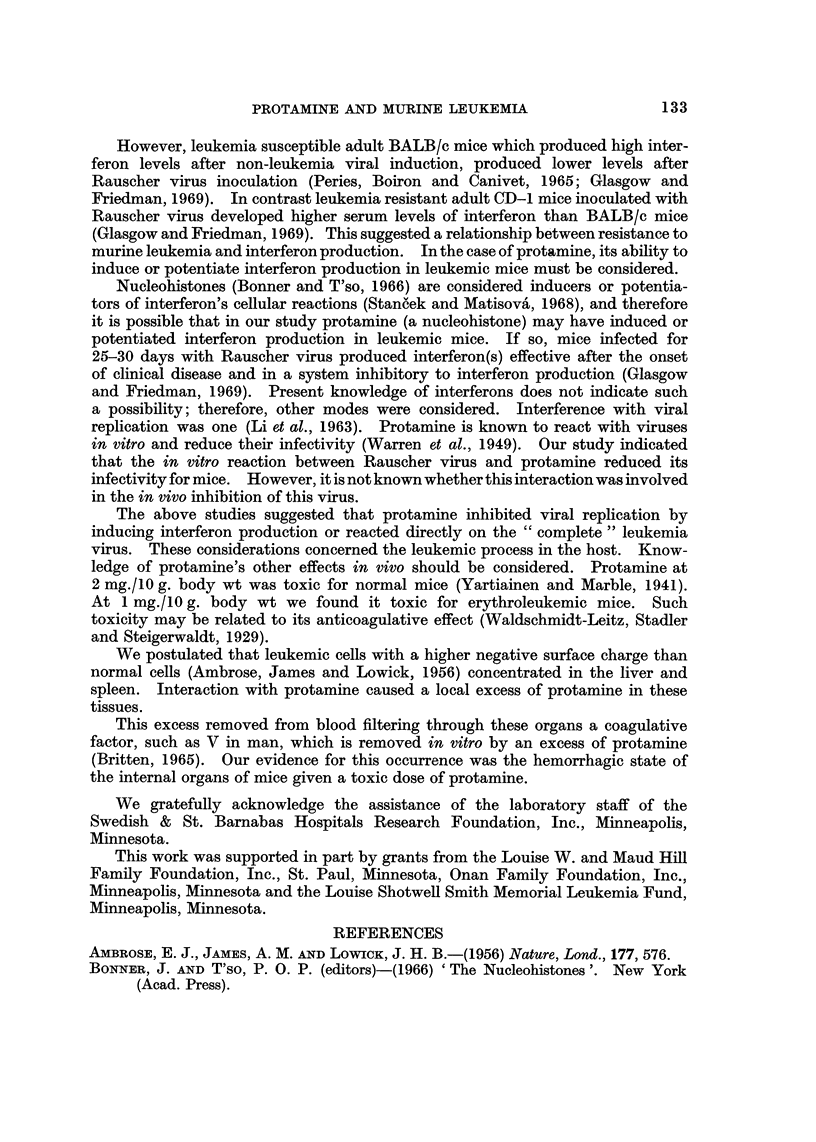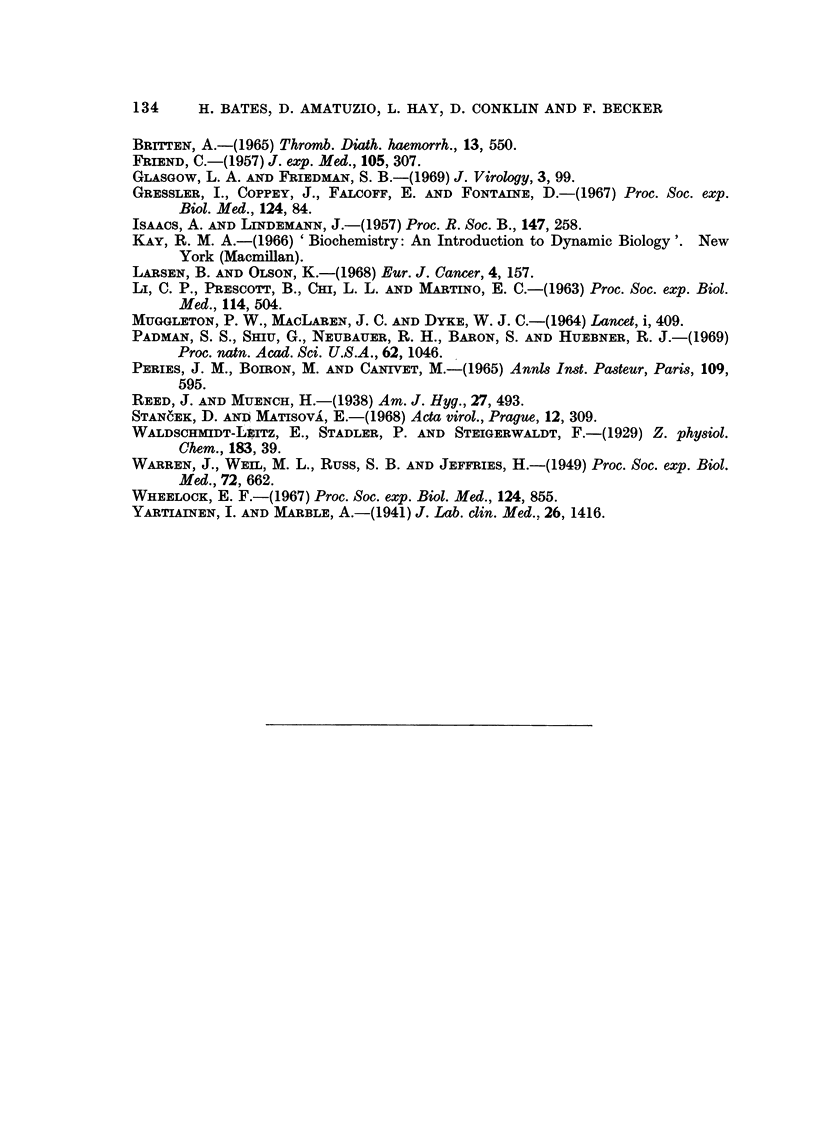# The Effects of Protamine on a Murine Leukemia Virus

**DOI:** 10.1038/bjc.1971.18

**Published:** 1971-03

**Authors:** H. A. Bates, D. S. Amatuzio, L. Hay, Dorothy J. Conklin, Francoise B. Becker

## Abstract

This study indicated that: (1) i.p. inoculation of protamine into (Rauscher) leukemic mice increased their *X* death time, (2) protamine was more toxic for leukemic than normal mice and (3) the *in vitro* reaction between Rauscher virus and protamine reduced its infectivity for mice.


### THE EFFECTS OF PROTAMINE ON A MURINE LEUKEMIA VIRUS

H. A. BATES, D. S. AMATUZIO, L. HAY, DOROTHY J. CONKLIN AND FRANCOISE B. BECKER

*From the Research Laboratory, Swedish and St. Barnabas Hospitals Research Foundation, Inc., Minneapolis, Minnesota, U.S.A.*

Received for publication October 29, 1970

**SUMMARY.—This study indicated that: (1) i.p. inoculation of protamine into (Rauscher) leukemic mice increased their $\bar{X}$ death time, (2) protamine was more toxic for leukemic than normal mice and (3) the *in vitro* reaction between Rauscher virus and protamine reduced its infectivity for mice.**

PROTAMINE complexes with nucleic acid in the sperm of salmon (salmine) and other fish (Kay, 1966). In mice it inhibited the growth of Landschutz ascites and sarcoma 180 tumors (Muggleton, MacLaren and Dyke, 1964). However, *in vitro* incubation of N.J.A. leukemic cells with protamine before inoculation into mice failed to inhibit the development of leukemia (Larson and Olson, 1968). Our study investigated what effects protamine had on Rauscher virus induced murine leukemia and, in a separate experiment, Rauscher virus infectivity in mice after *in vitro* incubation with protamine.

### MATERIALS AND METHODS

In the following experiments all dilutions were made in phosphate (0·01M) buffered saline (0·15M) pH 7·4 (PBS) and BALB/c/Tex inbred mice were utilized.

### In vivo *studies*

Day-old mice were inoculated intraperitoneally (i.p.) with 0·05 of a suspension containing 50–100 $MLD_{50}$ of Rauscher leukemia virus ($MLD_{50}$ = minimum concentration of virus killing 50% of test mice). When post inoculation mortality and hepatosplenomegaly were present, mice were divided into subgroups. Then leukemic mice in a subgroup were each inoculated i.p. three times per week for 6 weeks with one of the following concentrations of protamine sulfate/10 g. of body weight: (1) 0·2 mg., (2) 0·4 mg., (3) 0·6 mg. and (4) 1·0 mg. Control mice, the same age, consisted of: (1) normal noninoculated, isolated, (2) normal, isolated and inoculated with 1·0 mg. of protamine as described above, and (3) leukemic (viral controls). All mice were observed up to 120 days with mortality recorded daily and necropsies done on those which expired.

### In vitro *studies*

Rauscher virus and protamine were mixed in such a manner that the viral concentration/0·05 ml. was 50–100 $MLD_{50}$ and protamine in one of the following

* Reprint requests: Henry A. Bates, Ph.D., Metropolitan Medical Center, 900 South 8th Street, Minneapolis, Minnesota, 55404.

concentrations: (1) 0·025 mg., (2) 0·05 mg., (3) 0·1 mg. The mixtures were reacted at 20° C. for 30 minutes. Each was then centrifuged at 25,000 r.p.m. for 15 minutes at 4° C. The supernatant was decanted off and recentrifuged. The second and first sediments were pooled. These pooled sediments and the second supernatant were diluted with PBS to the original volume. Then undiluted, $10^{-1}$, $10^{-2}$ and $10^{-3}$ concentrations were prepared in PBS and each inoculated i.p. (0·05 ml.) into a group of day-old mice. Controls consisted of day-old mice: (1) inoculated i.p. with Rauscher virus (centrifuged and prepared as described above) incubated, (2) inoculated i.p. with 0·1, 0·05 or 0·025mg. of protamine and (3) normal mice held 60 days for splenic comparison. Mice were observed for 60 days and necropsies done on those which expired 48 hours after inoculation. Mortality after this period was used for determining $MLD_{50}$ values. Survivors at 60 days were necropsied and the presence of splenomegaly recorded (Friend, 1957). Splenomegaly and mortality were used in determining $SD_{50}$ values ($SD_{50}$ = minimum concentration of virus inducing splenomegaly in 50% of tested mice). Preliminary studies showed that spleens of 60-day-old normal mice weighed 250 ± 50 mg.; therefore, spleens over 300 mg. were considered pathological and used in $SD_{50}$ calculations.

### RESULTS

The maximum concentration of protamine sulfate used (Table I) was determined from a study which indicated that 1 mg. of protamine sulfate/10 g. body weight given i.p. was toxic for erythroleukemic mice. These mice expired 1–4 hours after injection. At necropsy, their livers, spleens and lymph nodes were necrotic and hemorrhagic. Mortality or similar lesions were not evident in normal mice.

TABLE I.—*Protamine Sulfate's Effect on Mean ($\bar{X}$) Death Time of Mice Infected with Viral (Rauscher) Leukemia*

| Group | Amount (mg.) | No. of mice | No. of expirations† | $\bar{X}$ Death (days)§ |
|---|---|---|---|---|
| Normals | — | 100 | 5/100‡ | N.S.M.‖ |
| Normals plus Protamine | 1·0 | 25 | 0/25 | N.S.M. |
| Leukemic* (viral controls) | — | 50 | 50/50 | 40 |
| Leukemic plus protamine | 0·2 | 40 | 40/40 | 50 |
| Leukemic plus protamine | 0·4 | 40 | 40/40 | 54 |
| Leukemic plus protamine | 0·6 | 30 | 28/30¶ | 78 |
| Leukemic plus protamine | 1·0 | 22 | 22/22 | 39 |

* Inoculated with Rauscher virus (no protamine).
† No. of expirations/No. of mice in group.
‡ Normal colony mortality over 3–5 months was 1–5%.
§ Calculated from day of viral inoculation (day 1) until death.
‖ N.S.M. = no significant mortality.
¶ 2 mice were still alive at 120 days.

At necropsy, the viral controls showed enlarged spleens, livers and ascites. Deaths occurred 30–45 days post inoculation with a $\bar{X}$ death time of 40 days. Leukemic mice inoculated with 0·2 or 0·4 mg. of protamine had greater survival times than the leukemic controls, but little evidence of tumor regression. Whereas, leukemic mice inoculated with 0·6 mg. of protamine showed little ascites after 2 weeks of inoculation and after 4–6 weeks post inoculation the hepatosplenomegaly had regressed. Mice which survived 120 days had no gross evidence of leukemia; however, microscopic examination of the spleens, livers and lymph nodes showed the presence of leukemic cells.

TABLE II.—*Results Obtained when the Rauscher Virus was Incubated* In Vitro *at 20° C. for 30 Minutes with Various Concentrations of Protamine*

| | Protamine (mg.)* | | | | | |
|---|---|---|---|---|---|---|
| | 0·025 | | 0·05 | | 0·1 | |
| Specimen | $MLD_{50}$ | $SD_{50}$ | $MLD_{50}$ | $SD_{50}$ | $MLD_{50}$ | $SD_{50}$ |
| Supernatant | 41† | 120 | 11 | 39 | 1 | 10 |
| Sediment | 10 | 24 | 10 | 31 | 1 | 10 |

| Controls | $MLD_{50}$ | $SD_{50}$ |
|---|---|---|
| 1. Virus—no incubation | 62 | 210 |
| supernatant | 62 | 210 |
| sediment | 1 | 10 |
| 2. Virus—incubation | — | — |
| supernatant | 56 | 190 |
| sediment | 1 | 10 |
| 3. Protamine—alone | — | — |
| 0·1 mg. | 0 | 0 |
| 0·05 mg. | 0 | 0 |
| 0·025 mg. | 0 | 0 |

* Concentration of protamine/0·05 ml. incubated with 50–100 $MLD_{50}$ of virus.

† Values are the reciprocal of dilutions containing 1 $MLD_{50}$ or 1 $SD_{50}$, *i.e.* number of $MLD_{50}$ or $SD_{50}$ in undiluted specimens. Calculations were based upon a standard method (Reed and Muench, 1938).

Preliminary studies indicated that *in vitro* incubation of virus and protamine at 20° C. for 30 minutes gave the most reproducible results, and therefore, was used in this study. The concentrations of protamine reacted *in vitro* with the Rauscher virus were based upon weight ratios (average weight of newborn—1 g.), and approximated those described in Table I. The average weight of spleens used for $SD_{50}$ calculations was 650 mg. with a range of 315–2200 mg. Necropsies on the viral control mice and those inoculated with virus and protamine which expired before 60 days showed gross lesions similar to those described under Table I. Control mice inoculated with 0·1 mg., 0·05 mg. or 0·025 mg. of protamine showed no mortality or evidence of hepatosplenomegaly.

### DISCUSSION

Our study indicated that intraperitoneal inoculation of protamine into leukemic mice prolonged their $\bar{X}$ death time and the *in vitro* reaction of protamine and the Rauscher murine leukemia virus reduced its infectivity for mice. The nature of neither inhibition was determined. Studies by others suggested possible modes of action.

One inhibitor of murine leukemia viruses is interferon (Isaacs and Linderman, 1957). Administered passively, it inhibited the *in vivo* development of viral (Friend) murine leukemia (Gressler *et al.*, 1967). These viruses are also inhibited *in vivo* by treatment with interferon inducing synthetic products (Padman, *et al.*, 1969) or statalon (Wheelock, 1967).

However, leukemia susceptible adult BALB/c mice which produced high interferon levels after non-leukemia viral induction, produced lower levels after Rauscher virus inoculation (Peries, Boiron and Canivet, 1965; Glasgow and Friedman, 1969). In contrast leukemia resistant adult CD–1 mice inoculated with Rauscher virus developed higher serum levels of interferon than BALB/c mice (Glasgow and Friedman, 1969). This suggested a relationship between resistance to murine leukemia and interferon production. In the case of protamine, its ability to induce or potentiate interferon production in leukemic mice must be considered.

Nucleohistones (Bonner and T'so, 1966) are considered inducers or potentiators of interferon's cellular reactions (Stanček and Matisová, 1968), and therefore it is possible that in our study protamine (a nucleohistone) may have induced or potentiated interferon production in leukemic mice. If so, mice infected for 25–30 days with Rauscher virus produced interferon(s) effective after the onset of clinical disease and in a system inhibitory to interferon production (Glasgow and Friedman, 1969). Present knowledge of interferons does not indicate such a possibility; therefore, other modes were considered. Interference with viral replication was one (Li *et al.*, 1963). Protamine is known to react with viruses *in vitro* and reduce their infectivity (Warren *et al.*, 1949). Our study indicated that the *in vitro* reaction between Rauscher virus and protamine reduced its infectivity for mice. However, it is not known whether this interaction was involved in the *in vivo* inhibition of this virus.

The above studies suggested that protamine inhibited viral replication by inducing interferon production or reacted directly on the " complete " leukemia virus. These considerations concerned the leukemic process in the host. Knowledge of protamine's other effects *in vivo* should be considered. Protamine at 2 mg./10 g. body wt was toxic for normal mice (Yartiainen and Marble, 1941). At 1 mg./10 g. body wt we found it toxic for erythroleukemic mice. Such toxicity may be related to its anticoagulative effect (Waldschmidt-Leitz, Stadler and Steigerwaldt, 1929).

We postulated that leukemic cells with a higher negative surface charge than normal cells (Ambrose, James and Lowick, 1956) concentrated in the liver and spleen. Interaction with protamine caused a local excess of protamine in these tissues.

This excess removed from blood filtering through these organs a coagulative factor, such as V in man, which is removed *in vitro* by an excess of protamine (Britten, 1965). Our evidence for this occurrence was the hemorrhagic state of the internal organs of mice given a toxic dose of protamine.

We gratefully acknowledge the assistance of the laboratory staff of the Swedish & St. Barnabas Hospitals Research Foundation, Inc., Minneapolis, Minnesota.

This work was supported in part by grants from the Louise W. and Maud Hill Family Foundation, Inc., St. Paul, Minnesota, Onan Family Foundation, Inc., Minneapolis, Minnesota and the Louise Shotwell Smith Memorial Leukemia Fund, Minneapolis, Minnesota.

### REFERENCES

Ambrose, E. J., James, A. M. and Lowick, J. H. B.—(1956) *Nature, Lond.*, **177**, 576.

Bonner, J. and T'so, P. O. P. (editors)—(1966) ' The Nucleohistones '. New York (Acad. Press).

BRITTEN, A.—(1965) *Thromb. Diath. haemorrh.*, **13**, 550.
FRIEND, C.—(1957) *J. exp. Med.*, **105**, 307.
GLASGOW, L. A. AND FRIEDMAN, S. B.—(1969) *J. Virology*, **3**, 99.
GRESSLER, I., COPPEY, J., FALCOFF, E. AND FONTAINE, D.—(1967) *Proc. Soc. exp. Biol. Med.*, **124**, 84.
ISAACS, A. AND LINDEMANN, J.—(1957) *Proc. R. Soc.* B., **147**, 258.
KAY, R. M. A.—(1966) ' Biochemistry: An Introduction to Dynamic Biology '. New York (Macmillan).
LARSEN, B. AND OLSON, K.—(1968) *Eur. J. Cancer*, **4**, 157.
LI, C. P., PRESCOTT, B., CHI, L. L. AND MARTINO, E. C.—(1963) *Proc. Soc. exp. Biol. Med.*, **114**, 504.
MUGGLETON, P. W., MACLAREN, J. C. AND DYKE, W. J. C.—(1964) *Lancet*, i, 409.
PADMAN, S. S., SHIU, G., NEUBAUER, R. H., BARON, S. AND HUEBNER, R. J.—(1969) *Proc. natn. Acad. Sci. U.S.A.*, **62**, 1046.
PERIES, J. M., BOIRON, M. AND CANIVET, M.—(1965) *Annls Inst. Pasteur, Paris*, **109**, 595.
REED, J. AND MUENCH, H.—(1938) *Am. J. Hyg.*, **27**, 493.
STANČEK, D. AND MATISOVÁ, E.—(1968) *Acta virol., Prague*, **12**, 309.
WALDSCHMIDT-LEITZ, E., STADLER, P. AND STEIGERWALDT, F.—(1929) *Z. physiol. Chem.*, **183**, 39.
WARREN, J., WEIL, M. L., RUSS, S. B. AND JEFFRIES, H.—(1949) *Proc. Soc. exp. Biol. Med.*, **72**, 662.
WHEELOCK, E. F.—(1967) *Proc. Soc. exp. Biol. Med.*, **124**, 855.
YARTIAINEN, I. AND MARBLE, A.—(1941) *J. Lab. clin. Med.*, **26**, 1416.